# Examining the drivers of agricultural carbon emissions in Africa: an application of FMOLS and DOLS approaches

**DOI:** 10.1007/s11356-023-25173-8

**Published:** 2023-03-15

**Authors:** Talent Thebe Zwane, Thomas Bilaliib Udimal, Lariba Pakmoni

**Affiliations:** 1 School of Economics, University of Johannesburg, Johannesburg, South Africa; 2 School of Economics and Management, Southwest Forestry University, Kunming, China; 3 School of Public Policy, Chiang Mai University, Chiang Mai, Thailand

**Keywords:** Agricultural carbon emissions, Agriculture growth, Renewable energy consumption, Fertiliser consumption

## Abstract

The major aim of this study was to investigate the impact of renewable energy consumption and agricultural economic growth on agricultural carbon emissions in Africa for the period 1990–2019. This paper employed panel fully modified ordinary least square (PFMOLS) and dynamic ordinary least square (PDOLS) estimation techniques. The empirical results showed that agriculture growth promote agricultural carbon emissions. More precisely, the results revealed a strong positive and statistical significant impact on agricultural carbon emissions in Africa. On the other hand, the results on quadratic show a negative causal association between agriculture growth and agricultural carbon emissions in Africa. Interestingly, renewable energy consumption was found to have a negative impact on agricultural carbon emissions. On Granger causality, the result shows that there is a unidirectional causality between agriculture growth and agricultural carbon emissions. Based on these findings, we recommend that countries should promote and encourage the use of renewable energy to curb agricultural carbon emissions. There is a need to adopt agricultural practices that have the potential to limit carbon emissions.

## Introduction

Achieving balanced economic growth without compromising the environment is key to sustainable development. The rapid development attributable to industrialisation has hastened the pace of the global warming that is a threat to mankind and the entire world. The average surface temperature, according to the Fifth Assessment Report of the Intergovernmental Panel on Climate Change (Edenhofer 2015), increased by 0.85 °C between 1880 and 2012. Global warming is mainly caused by greenhouse gas (GHG) emissions. The Kyoto Protocol was passed to help minimise the rate of these emissions and became the first regulation aimed at minimising GHG emissions.

In his most recent study, Lindvall (2021) argued that the impact of global warming on global food production is expected to be higher by the close of the twenty-first century. This will make it difficult to feed a growing population, especially in Africa, where the population is expected to increase at a higher rate. The development will be a threat to fragile democratic systems and lead to chaos. Without global warming mitigation initiatives, the real domestic product (GDP) per capita is estimated to decrease by more than 7% by 2100 (Kahn et al. 2021). However, adopting the migration measures proposed in the Paris Agreement would result in only about a 1% loss in GDP. Jevrejeva et al. (2018) put the annual cost of global warming at US$1.7 trillion, attributable to the rise in sea levels, which is 2% of the global GDP.

Agriculture remains a major contributor to greenhouse gas emissions. As a result, agriculture’s contribution to global warming requires critical attention. In agriculture and other human-related activities, GHG emissions have been estimated at 70% of N_2_O, 20% of CO_2_ and 50% of CH_4_ (IPCC 1996). Agriculture contributes substantially to GHG emissions through livestock, rice production and the use of fertiliser (Vermeulen et al. 2012; Smith et al. 2007; Barker et al. 2007). The agriculture sector also contributes indirectly to emissions through land-use conversion (van der Werf et al. 2009; Vermeulen et al. 2012). The FAO estimates have put greenhouse gas emissions from sub-Saharan Africa low compared to other parts of the world (Hijbeek et al. 2020). However, with the increasing demand for food, GHG emissions from agriculture are expected to increase in sub-Saharan Africa (Smith et al. 2007). Population increases and changes in dietary requirements are projected to increase the demand for cereals three times by 2050 relative to 2010 (Van Ittersum et al. 2016). The entire continent has already started experiencing an increase in emissions, mostly attributable to deforestation, of which agriculture is the main cause (Curtis et al. 2018; Carter et al. 2017).

The Paris COP21 Agreement was adopted by 195 nations and aimed at keeping global warming below 2 °C, with initiatives to bring it to the pre-industrial level of 1.5 °C (IPCC V 2018). In addition, the sustainable development goals emphasise that climate change mitigation will have to go hand-in-hand with achieving food security (Ban 2016). However, it is argued that the effects of climate change mitigation on food security in sub-Saharan Africa (SSA) will be higher than the effect of climate change itself (Hasegawa et al. 2018). In recent times, food production in SSA has experienced a significant expansion of area, but with low yields (Hijbeek et al. 2020). The development has led to an increase in GHG emissions attributable to the land-use change (Bennetzen et al. 2016). To reduce GHG emissions and maintain biodiversity, agricultural intensification on the existing farmlands is often recommended (Cassman et al. 2003) because intensified agriculture has the lowest emission per unit of product produced (Bennetzen et al. 2016).

With the growing demand for food and calls to boost agricultural production to meet rapid increasing population, especially in sub-Saharan Africa, more agricultural lands will be put under cultivation resulting in an increase use of fertiliser (Wang and Dong 2019; IPCC 2014) (This development is likely to cause an increase in the level of greenhouse emissions. Even though nutrient inputs are currently low in SSA, intensification is likely to increase fertiliser input substantially (Ten Berge et al. 2019). An increase in fertiliser application, either organic or inorganic, is expected to increase emissions of GHG (Palm et al. 2010), and the inefficient use of soil nutrients in Africa presents a higher potential increase in the rate of GHG emissions (Reay et al. 2012).

Climate change is associated with rise in temperatures resulting in an increase in the levels of carbon dioxide, which impact is expected to vary sharply between developing and developed countries (Reilly et al. 2001; Darwin and Kennedy 2000). The drivers of agricultural response to climate change include biophysical and socio-economic factors. Agricultural activities led to a rise in temperatures, change is precipitation regimes, and an increase in greenhouse gas emissions. Agricultural activities such as land use change, fertiliser application are reported to contribute to changes in soil carbon (C), nitrogen (N) dynamics and ultimately atmospheric GHG emissions (Sun et al. 2016; Ogle et al. 2014). Compared to other sectors, greenhouse gas emissions in agricultural sector have not received much attention among scholars (Rajaniemi et al. 2011).

It is against this backdrop this study seeks to examine the drivers agricultural carbon emissions specifically in Africa. To help achieve the objective of the study, the study specifically looks at how fertiliser consumption, renewable energy consumption, growth in agriculture drive agricultural GHG emissions. The study will guide policy makers in the formulation of policies to curb GHG emissions emanating from agricultural sector with key emphasis on some key areas.

**Fig. 1:**
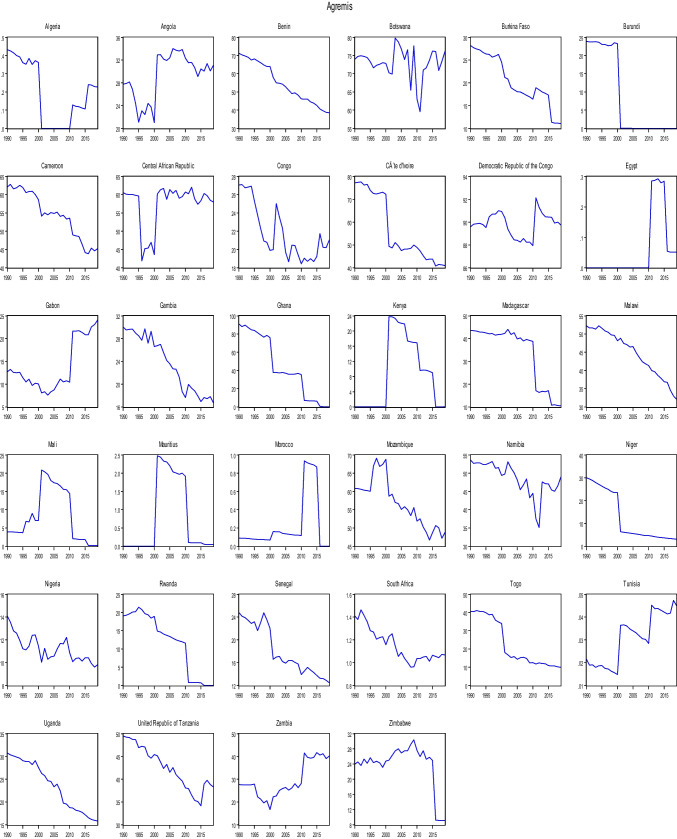
Trends in agricultural emissions

Figure 1 in the Appendix shows the patterns of agricultural emissions for 34 African countries between 1990 and 2019: Whereas some countries have experienced a consistent decline in the rate of agricultural carbon emissions, others have experienced an increase. Some have experienced a rise and fall in the agricultural carbon emissions trends. Generally, the pattern of agricultural carbon emissions has not been smooth. There has been intermittent rise and fall for almost all countries. The development calls for empirical examination of the impact of agricultural growth, fertiliser consumption and renewable energy use on the rate of carbon emissions. To this end, the study relied on non-linear approaches to examine the long-run effect of these three elements on agricultural carbon emissions.

## Literature review

The causal nexus between energy consumption and economic growth has received greater attention since the oil crisis in 1970. However, the test results regarding the direction of the impact of these variables have been mixed and inconsistent. The conflicting findings might be because different datasets were used and different periods were covered. Another contributing factor might be that previous studies in this field have relied heavily on bivariate techniques, which, according to Stern (1993), fail to detect causality due to omitted variables bias and the substitution effects that may occur between variables of interest such as energy and other inputs.

In the literature, there are four main hypotheses to explain the association between energy consumption and economic growth. The first hypothesis, called the ‘growth hypothesis’, postulates that there is a unidirectional causal association between energy consumption and economic growth (Yu and Choi 1985). It assumes that any limitation on energy consumption will have a negative effect on economic development (Inglesi-Lotz 2016; Ouyang and Li 2018). The second hypothesis, termed the ‘conservation hypothesis’, was propounded by Kraft and Kraft (1978) and claims that there is a unidirectional causal nexus between economic growth and energy consumption, suggesting that formulating and implementing conservation energy consumption strategies could have no significant adverse impact on economic growth (Mehrara 2007; Ouedraogo 2013). The third hypothesis is the ‘neutrality hypothesis’ (Akarca and Long 1980), which states that there is no causality between energy consumption and economic growth (Menegaki 2011; Alper and Oguz 2016). The first empirical evidence to support this hypothesis was provided by Akarca and Long (1980), who were later supported by Yu and Choi (1985). The last hypothesis, referred to as the ‘feedback hypothesis’, suggests that there is a bidirectional causal association between energy consumption and economic growth. Erol and Yu (1987) presented empirical evidence for this hypothesis from industrialised countries such as Canada, France, Italy, Japan and the UK. The hypothesis states that economic outputs and energy usage are mutually determined (Appiah 2018; Ha et al. 2018).

Empirical studies of the causal relationship between energy consumption and economic growth have found evidence to support a unidirectional causality running from economic growth to energy consumption. Such studies include the work of Souhila and Kourbali (2012) for Algeria, Zhang and Cheng (2009) for China, Adom (2011) for Ghana, Vidyarthi (2013) for India, Mohammed Albiman et al. (2015) for Tanzania, Kraft and Kraft (1978) for the USA, and Tang et al. (2016) for Vietnam.

The pioneering work of Kraft and Kraft (1978) investigated the link between energy consumption and economic growth in the USA for the period 1947 to 1974. The empirical findings from Sims Granger causality analysis reported a unidirectional causality running from gross national product to energy consumption for the USA. The authors suggested that energy conservation strategies could be introduced without hurting economic growth in the USA. Following the work of Kraft and Kraft (1978), Zhang and Cheng (2009) examined the presence and direction of Granger causality between economic growth, energy consumption, and carbon emissions in China. Using data covering the period 1960 to 2007, the authors reported a unidirectional Granger causality flowing from GDP to energy consumption, and a unidirectional Granger causality flowing from energy consumption to carbon emissions in the long run.

In an unrelated empirical work, Vidyarthi (2013) assessed a long-term and causal association between energy consumption, economic growth and carbon emissions in India, employing data covering 1971 to 2009. The author applied the Johansen cointegration model to establish the cointegration between the chosen factors, and used the vector error correction model (VECM) Granger causality test to determine the direction of causality. The results of the Johansen cointegration analysis determined a long-term linkage between energy consumption, carbon emissions and economic growth. The long-term causality findings supported a unidirectional causality from energy consumption and CO_2_ emissions to economic growth, while the short-term causality showed mixed findings. Thus, unidirectional causality runs from energy consumption to carbon emission, from carbon emission to economic growth, and from economic growth to energy consumption (Vidyarthi 2013).

A study by Mohammed Albiman et al. (2015) examined the causal nexus between energy consumption, environmental pollution and per capita economic growth in Tanzania. Using data spanning 1975 to 2013, the authors implemented the more robust causality technique of Toda and Yamamoto’s non-causality test. The results showed a unidirectional causality running from economic growth and energy consumption to environmental pollution through CO_2_ emissions (Mohammed Albiman et al. 2015).

Tang et al. (2016) assessed the long-run association between energy consumption and economic growth in Vietnam using data covering the period between 1971 and 2011. Implementing Granger causality, Tang et al. (2016) reported a one-way causality flowing from energy consumption to economic growth. The authors concluded that Vietnam, as an energy-intensive nation, needs to apply renewable energy strategies to provide an adequate supply of energy because this will boost economic growth.

Several studies have endorsed a bidirectional causality, such as those of Al-mulali et al. (2015) for emerging countries, Apergis and Payne (2009) for 11 countries of the Commonwealth of Independent States, Belke et al. (2011) for 25 OECD Countries, Gokmenoglu and Taspinar (2018) for Pakistan, Khobai and Le Roux (2017) for South Africa, Lee and Lee (2010), Osigwe and Arawomo (2015) for Nigeria, Ozturk et al. (2010) for lower-middle income, Pao et al. (2014) for Brazil, Saidi and Hammami (2015) for Tunisia, Rezitis and Ahammad (2015) for South and Southeast Asian countries, Vafaeirad et al. (2015) for seven Asian countries, and Liu et al. 2019 for China, India and G7 countries.

Saidi and Hammami (2015) used Tunisia as a laboratory testing ground and employed annual data covering the period 1974 to 2011. Johansen model results established a long-run linkage between economic growth and energy consumption in the study area, while the Granger causality results revealed a bidirectional causality between energy consumption and economic growth.

Khobai and Le Roux (2017) contributed to the most recent studies by assessing the association between energy consumption, economic growth, and carbon dioxide emission in South Africa from 1971 to 2013. Their Johansen test of cointegration revealed a long-run association between energy consumption, CO_2_ emissions, economic growth, trade openness and urbanisation. Their findings for the existence and direction of vector error correction model (VECM) Granger causality showed a bidirectional causality between energy consumption and economic growth in the long run (Khobai and Le Roux 2017). Furthermore, the VECM estimates showed a unidirectional causality running from CO_2_ emissions, economic growth, trade openness and urbanisation to energy consumption, and from energy consumption, CO_2_ emissions, trade openness and urbanisation to economic growth (Khobai and Le Roux 2017). These authors argued that their findings suggest a new perspective on establishing energy strategies that can improve economic growth in South Africa.

Gokmenoglu and Taspinar (2018) assessed the long-run equilibrium linkage between CO_2_ emissions, income growth, energy consumption, and agriculture in Pakistan between 1971 and 2014. The results confirmed a bidirectional causal nexus between gross domestic product, energy use, agriculture and CO_2_ emissions. Moreover, the results of the fully modified ordinary least squares model revealed that GDP had an elastic positive effect on CO_2_ emissions, while energy use and agricultural value added had an inelastic positive influence on CO_2_ emissions, although the squared GDP had an inelastic and negative impact on CO_2_ emissions (Gokmenoglu and Taspinar 2018).

Implementing the bounds testing to cointegration technique in a multivariate estimator with carbon emissions, energy use, income, and foreign trade, Halicioglu (2009) reported a bidirectional Granger causality (in both the short and the long run) between carbon emissions and income in Turkey.

Jebli and Youssef (2017) used the panel cointegration approach and Granger causality to examine the dynamic causality between AVA, per capita renewable energy consumption, real GDP and CO_2_ emissions. The results showed a short-run bidirectional causality between agriculture and carbon emissions. The study further established a long-run bidirectional between renewable energy and carbon emissions. Liu et al. (2019) applied multispatial convergent cross mapping to analyse energy-carbon-economy relationship for China, India and the G7 countries. Interestingly, Liu et al (2019) revealed a bidirectional relationship between energy consumption, carbon emissions and economic growth in China and India, while numerous causal associations were noted for the G7 countries, including bidirectional, unidirectional and neutral nexus. In their recent paper, Xu et al. (2020) this paper selects 2001 to 2016 panel data of China’s 30 provinces, and applied nonparametric additive regression models to investigate the agricultural sector’s CO_2_ emissions. The empirical findings showed that economic growth, financial capacity, and energy intensity all have a “pull first, then restrict” inverted U-shaped nonlinear effect on CO_2_ emissions in China.

On the other hand, a limited number of studies have found no causality between energy consumption and economic growth. Such empirical studies included Jobert and Karanfil (2007), Ozturk and Acaravci (2010), and Çetin and Seker (2012) for Turkey. Kalyoncu et al. (2013) assessed the association between energy consumption and economic growth in Georgia, Azerbaijan and Armenia during the period 1995–2009. They implemented Engle–Granger cointegration and Granger causality tests, and the results revealed no causality between the two previously mentioned variables for Georgia. Likewise, Eden and Jin (1992) concluded that there is no causality between energy consumption and economic growth in the USA.

Lin and Xu (2018) investigated the factors affecting CO_2_ emissions in China’s agriculture sector employing quantile regression. Interestingly, the results showed that the impact of economic growth on CO_2_ emissions in the upper 90th and 75th–90th quantile provinces were higher than in the 50th–75th, 25th–50th, 10th–25th and lower 10th quantile provinces due to the differences in fixed–asset investment and agricultural processing (Lin and Xu 2018). The author found that the effect of energy efficiency in the upper 90th, 75th–90th, and 50th–75th quantile provinces were stronger than those in the 25th–50th, 10th–25th, and lower 10th quantile provinces because of the huge difference in R&D funding and R& D personnel investments (Lin and Xu 2018).

Notwithstanding the contributions of the various studies conducted on this topic, empirical studies involving the relationship between carbon emissions, economic growth and energy consumption in the agriculture sector are scant for Africa. In fact, studies on carbon emissions have concentrated mainly on developed economies and have not placed much emphasis on the African continent. Because of the increasing relevance of African countries’ contribution to climate change, their involvement in the global problem–solution approach has become paramount (Pittel and Rübbelke 2008). Africa’s growing population and increasing demand for food necessitate an examination of its agricultural emissions since it is an agriculture-dependent economy. Hence, the driving objective of this study was to investigate the impact of renewable energy consumption, fertiliser consumption and agricultural economic growth on agricultural carbon emissions in 34 African countries.

## Data and methodology

We relied on the statistics of the Food and Agriculture Organisation (FAO) and World Bank to analyse data for 34 African countries from 1990 to 2019. The variables included in the study were agricultural carbon emissions (AgricEm), fertiliser consumption (F), renewable energy consumption (REC) and agriculture’s share of GDP (AgricGDP). The selection of countries for the study was based on the availability of data on the variables under consideration.

Table 1 shows the variables and their respective modes of measurement.

**Table 1:** Variables and description of measurement

Variables	Mode of measurement	Source
Agricultural carbon emissions	Land use change Emissions share (CO_2_ eq % of total emissions)	FAOSTATS DATA
Fertiliser consumption	Kilogrammes per hectare of arable land	World Bank database
Renewable energy consumption	Per centage of total final energy consumption	World Bank database
Agricultural share of GDP	AgrGDP = Annual growth $US, 2015 prices	World Bank database

### Empirical model specification

In this study, we adopted the fully modified ordinary least squares (FMOLS) model to examine the relationship between agricultural carbon emissions, renewable energy consumption fertiliser consumption and agriculture’s share of GDP in Africa. The econometric model adopted was also used by Dogan and Seker (2016), Saboori and Sulaiman (2013), and Chandran and Tang (2013). However, the variables used in the current study differ from the variables used in those studies. An understanding of the relationship between agricultural carbon emissions (dependent variable), and fertiliser consumption, renewable energy consumption, and the sector’s share of GDP is required to formulate appropriate policies to curb emissions in the agriculture sector. The long-run relationship among the variables is given in the following equation:

1 $${AgricEm}_{it}={\pi }_{0t}+{\pi }_{1}{F}_{it}+{\pi }_{2}{REC}_{it}+{\pi }_{3}{AgricGDP}_{it} +{\varepsilon }_{it}$$

${AgricEM}_{it}$ refers to the agricultural carbon emissions at time $t$, and $i$ is the specific country; ${F}_{it}$ is the fertiliser consumption at time $t$, and the specific country is represented by $i$; ${REC}_{it}$ is the fertiliser consumption at time $t$, and for specific country is represented by $i$; ${AgricGDP}_{it}$ is the agriculture share of GDP at time $t$, and the specific country is represented by $i$. The beta coefficients ${\pi }_{1}$, ${\pi }_{2}$, and ${\pi }_{3}$ represent fertiliser consumption, renewable energy consumption and agriculture’s share of GDP, respectively. The data set was in panel form, so the long-run cointegration specifications stated by Perron (1989) and Panigrahi (2017) were employed. The error term ${\varepsilon }_{t}$ is assumed to be identically distributed.

### Econometric model

This paper presents some empirical analysis of the impact of agricultural economic growth and the consumption of renewable energy and fertiliser on agricultural carbon emissions in the selected number of African countries. The study implemented the dynamic ordinary least squared (DOLS) and the fully modified ordinary least squared (FMOLS) approach to determine their long-run relationship. We considered agricultural carbon emissions as the dependent variable while agricultural growth, fertiliser consumption and renewable energy consumption were explanatory variables. We begin our analysis by exploring, first, a panel root analysis to determine the stationarity of the variables. After non-stationarity was established, cointegration relationships among variables were established through the panel cointegration technique.

### Panel unit root tests

The panel unit root tests were used to determine the stationarity variable with the null hypothesis that there is a unit root. It was necessary to check for spurious correlations. The series was assumed under intercept, constant and trend. The equation for ‘without constant and trend’ was specified in Appendix 2.

According to Hoang and McNown (2006), the stationarity is achieved after differencing $(\Delta )$ of the series. The Levin and Lin (LL), Maddala Wu and Im-Pesaran-Shin (IPS) are the most widely used unit roots tests (Ucar and Omay 2009). However, LL has a setback in its application because it has an unrealistic hypothesis. The model developed by Im, Pesaran (Ucar and Omay 2009) is adopted for the study and stated below as:

2 $${y}_{i,t}={\beta }_{i}+{\gamma }_{i}{y}_{i.t-1}+{\varepsilon }_{i,t}$$

For all *i* = 1,…, N and *t* = 1, 2, 3,…, T.

We used the Barlett Kernel Method recommended by Phillips and Hansen (1990) to choose the optimal lag.

### The FMOLS estimator

The PFMOLS estimator was developed by Phillips and Hansen (Pedroni 2001) to estimate an optimal cointegrating regression. However, the study relied on the Pedroni (Hamit-Haggar 2012) heterogeneous PFMOLS estimator for the panel cointegration regression. The latter has the ability to deal with endogeneity bias and serial correlation (Mi et al. 2015). According to Pedroni (1996), PFMOLS is suitable for panel Hamit-Haggar, which has heterogenous cointegration, suggesting that a panel FMOLS estimator for the coefficient *β* of model 1 was:

3 $${\beta }_{NT}^{*}-\beta =\left(\sum\limits_{i=1}^{N}{L}_{22i}^{-2}\sum\limits_{i=1}^{T}{\left({x}_{it}-{\overline{x} }_{it}\right)}^{2}\right)\sum\limits_{i=1}^{N}{L}_{11i}^{-1}{L}_{22i}^{-1}\left(\sum\limits_{i=1}^{N}\left({x}_{it}-{\overline{x} }_{l}\right){\mu }_{it}^{*}-T{\widehat{\gamma }}_{l}\right)$$

where

$${\mu }_{it}^{*}={\mu }_{it}-\frac{{\widehat{L}}_{21i}}{{\widehat{L}}_{22i}} \Delta {x}_{it}, {\widehat{\gamma }}_{i}={\widehat{\Gamma }}_{21i}{\widehat{\Omega }}_{21i}^{0}-\frac{{\widehat{L}}_{21i}}{{\widehat{L}}_{22i}}\left({\widehat{\Gamma }}_{22i}\right.+{\widehat{\Omega }}_{22i}^{0},$$

and ${\widehat{L}}_{i}$ was the lower triangulation of ${\widehat{\Omega }}_{i}$.

The panel PFMOLS estimator had the same asymptotic distribution as that of the PDOLS estimation derived by Pedroni (Granger 1988). Both PDOLS and PFMOLS analyses were carried out for a robustness check of the results.

## Results

Before presenting the empirical results, it is important to present some basic descriptive analysis, so the summary statistics and the correlation matrix are presented in Tables 2 and 3, respectively. In Table 1, summary statistics and the measures of mean, median, standard deviation, maximum, minimum, kurtosis and skewness can be identified. What is notable in this table is that the highest mean is for REC at 64.42215 and the lowest mean is for AgricGDP at 20.95677. Almost all the variables have a skewness value of close to zero, which is an indication of normality. The kurtosis values are all less than 3, which implies the normality of the variables. The smaller values of Jarque–Bera probability show that parameters are normal.

**Table 2:** Descriptive statistics

	AgricEM	AgricGDP	F	REC
Mean	26.639	20.957	39.612	64.422
Median	21.736	20.609	9.803	77.874
Maximum	92.119	56.544	600.078	98.343
Minimum	0.0000	1.798	0.0000	0.059
Std Dev	24.088	11.848	95.86358	29.009
Skewness	0.815	0.285	0.898	− 0.904
Kurtosis	2.884	2.317	2.972	2.424
Jarque-Bera	1.096	2.952	1.871	3.316
Probability	0.475	0.744	0.540843	0.183
Sum	24,188.50	19,028.75	35,968.07	58,495.31
Sum Sq. Dev	526,285.0	127,318.8	8,335,173	763,273.6
Observations	908	908	908	908

**Table 3:** Correlations

	*F*	AgricEM	AgricGDP	REC
LF	1	− 0.309	− 0.287	0.522
LAgricEM	− 0.309	1	0.216	0.410
LAgricGDP	− 0.287	0.216	1	0.630
LREC	0.522	0.410	0.630	1

**Fig. 2:**
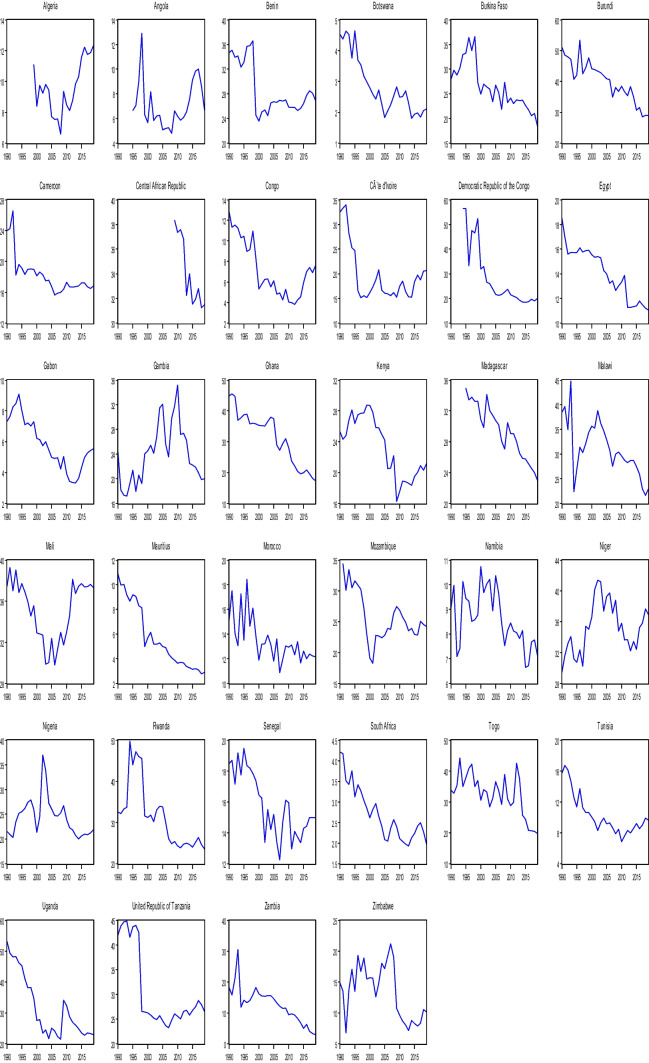
Trends in agriculture growth

**Fig. 3:**
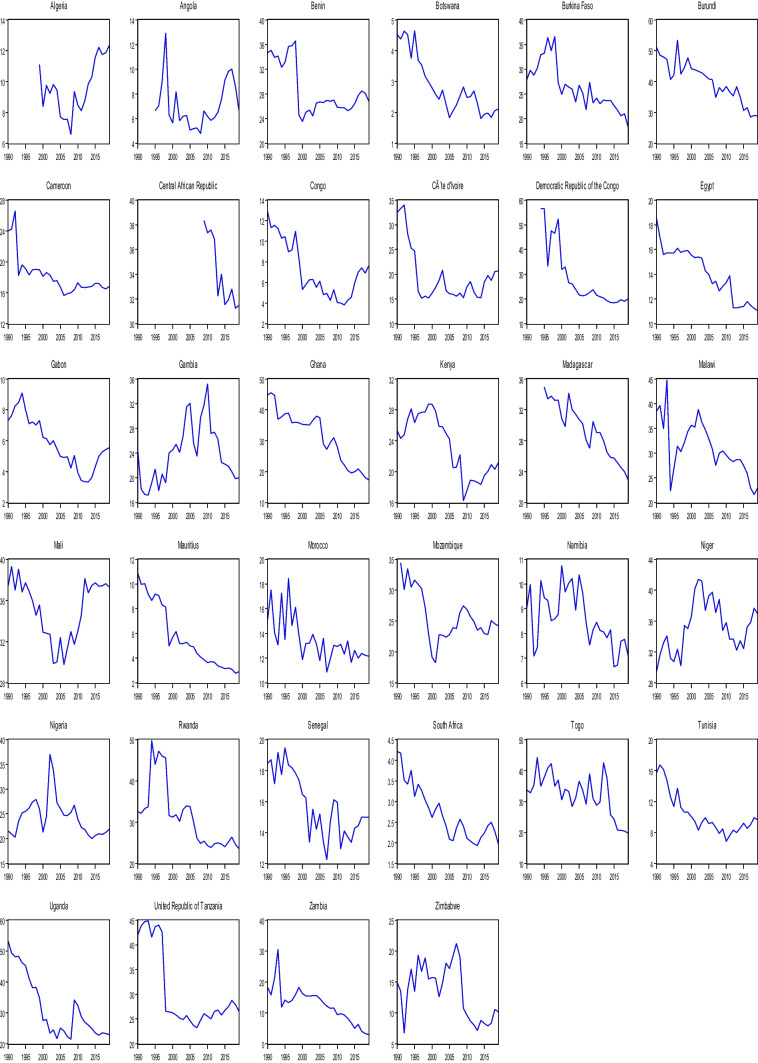
Trends in fertiliser consumption

**Fig. 4:**
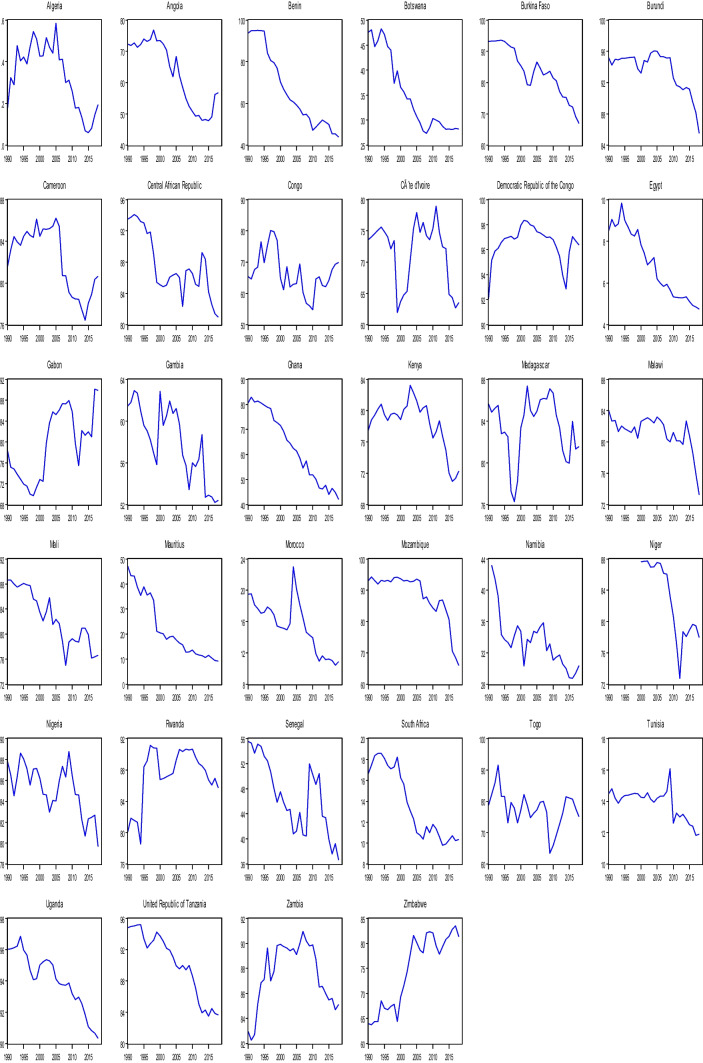
Trends in renewable energy use

In Table 3, LF is negatively related to AgricEM and AgricGDP, whereas they are positively correlated with REC. Figures 2, 3 and 4 in the Appendix show the trends of agricultural growth, fertiliser consumption and renewable energy use, respectively.

Table 4 presents the results of the panel unit root tests. The results at level, first difference and second difference show that there was no stationarity for any of the variables because the results were found to be statistically significant. Based on the results presented in this table, we could not accept the null hypothesis for the unit root.

**Table 4:** Panel unit root test

Variables	Level	1st difference	2nd difference
LAgricEM	2.135	− 25.315^***^	− 36.383^***^
LAgricGDP	− 2.101^**^	− 29.228^***^	− 35.809^***^
LF	3.646	− 29.371^***^	− 35.783^***^
LREC	2.968	− 22.032^***^	− 34.359^***^

Method: Im, Pesaran and Shin *W*-stat; null hypothesis: unit root (individual unit root process)

After establishing no stationarity in the variables, we checked for cointegration among agricultural carbon emissions, agriculture growth, fertiliser consumption and renewable energy consumption. As suggested by Arouri et al. (2012) and Hamit-Haggar (2012), the residual cointegration estimates presented in Table 5 satisfy the criteria. Most of the tests were statistically significant; therefore, we could not accept the null hypothesis that there is no cointegration. The results show that the variables were cointegrated at statistically significant levels under no deterministic trend.

**Table 5:** Pedroni residual cointegration test

Alternative hypothesis: common AR coefs. (within-dimension)				
	Statistic	Prob	Weighted statistic	Prob
Panel v-statistic	− 1.272	0.898	− 1.619	0.947
Panel rho-statistic	− 0.257	0.399	− 0.446	0.328
Panel PP-statistic	− 3.041^***^	0.001	− 2.999^***^	0.001
Panel ADF-statistic	− 2.677^***^	0.004	− 2.254^**^	0.012
Alternative hypothesis: individual AR coefs. (between-dimension)				
	Statistic	Prob		
Group rho-statistic	1.039	0.851		
Group PP-statistic	− 4.597^***^	0.000		
Group ADF-statistic	− 4.715^***^	0.000		

Null hypothesis: no cointegration; trend assumption: no deterministic trend; automatic lag length selection based on SIC with lags from 0 to 5; Newey-West automatic bandwidth selection and Bartlett kernel

Following suggestions by Arouri et al. (2012) and Hamit-Haggar (2012), the residual cointegration estimates presented in Table 6 satisfy the criteria. Most of the tests were statistically significant; therefore, we could not accept the null hypothesis that there is no cointegration. The result shows that the variables were cointegrated at statistically significant levels under no deterministic intercept or trend.

**Table 6:** Pedroni residual cointegration test

Alternative hypothesis: common AR coefs. (within-dimension)				
	Statistic	Prob	Weighted statistic	Prob
Panel v-Statistic	− 0.855	0.804	− 1.674	0.953
Panel rho-Statistic	− 1.768	0.039	− 1.049	0.147
Panel PP-Statistic	− 4.771^***^	0.000	− 3.642^***^	0.0001
Panel ADF-Statistic	− 3.650^***^	0.0001	− 2.505^***^	0.006
Alternative hypothesis: individual AR coefs. (between-dimension)				
	Statistic	Prob		
Group rho-Statistic	0.084	0.534		
Group PP-Statistic	− 4.887^***^	0.000		
Group ADF-Statistic	− 4.771^***^	0.000		

null hypothesis: no cointegration; trend assumption: no deterministic intercept or trend; automatic lag length selection based on SIC with lags from 1 to 6; Newey-West automatic bandwidth selection and Bartlett kernel

Table 7 presents the results on the PFMOLS model. The table has four numbered columns. Column 1 represents the PFMOLS results at none level, while column 2 reports the same model estimates at the constant levels. Column 3 shows the FMOLS estimates for linear trends, and column 4 represents the FMOLS results for quadratic trends. We also applied numerous diagnostic tests to ascertain the goodness of fit of the estimated models. First, the *R*^2^ adjusted *R*^2^ values improved across all the four models, implying that the estimated regression model fits quite well. The dependent variable is agricultural carbon emissions in logarithmic terms. The results of PFMOLS indicate that, when other factors are held constant, the predicted long-run estimates of agricultural growth are negative and insignificant in models 1 and 3. However, the coefficient is significant at the 1% level in model 4, indicating that a 1% increase in agricultural growth will result in a − 0.1354% reduction in carbon emissions. See also the work of Rahman and Alam (2022). The coefficient of agricultural growth is positive and statistically significant at the 1% level of significance in model 2, implying that increasing agricultural growth by 1% causes a 0.6228% increase in carbon emissions in the long run.

**Table 7:** Results from the panel fully modified ordinary least square approach

Variable	1. None	2. Constant level	3. Linear trend	4. Quadratic trend
Agricultural growth	− 0.184 (0.149)	0.623^***^ (0.097)	− 0.087 (0.067)	− 0.135^**^ (0.060)
Fertiliser consumption	− 0.010 (0.013)	0.003 (0.021)	0.024 (0.016)	− 0.004 (0.012)
Renewable energy consumption	− 0.437297^***^ (0.049653)	− 0.550^***^ (0.071)	− 0.118^***^ (0.029)	− 0.196^**^ (0.059)
R-squared	0.160	0.908	0.974	0.984
Adjusted R-squared	0.158	0.904	0.971	0.982
S.E. of regression	21.798	7.371	4.021	3.198
Long run variance	1581.679	147.418	34.063	16.521

Values in parenthesis are std errors: ***, **, and * at 1%, 5% and 10%, respectively

Source: authors’ calculation

Furthermore, the estimated long-run coefficient of fertiliser consumption is positive and insignificant in models 1 and 4, which reveals that fertiliser consumption by 1% is associated with a decrease in carbon emissions by − 0.0101% and − 0.0035% in the long run. Therefore, fertiliser consumption deteriorates agricultural emissions in Africa. However, fertiliser consumption has positive but insignificant coefficients in both model 2 and model 3, implying that increasing fertiliser consumption by 1% results in 0.0033% and 0.0240% increases in agricultural carbon emissions, respectively. See also the empirical work of Liu et al. (2019).

On the other hand, the estimated coefficients of renewable energy consumption are negative and significant at a 1% level, suggesting that increasing renewable energy consumption by 1% leads to − 0.437%, − 0.550%, − 0.117% and − 0.195% reduction in carbon emissions in models 1 to 4, respectively. These outcomes show the emission reduction potential of increasing the use of renewable energy in the African continent. These results confirm the estimates of Ulucak et al. (2021) and indirectly also those of Demircan et al. (2021), contributing to the discussion around the renewable energy consumption–agricultural carbon emissions nexus. In their recent empirical work, Rahman and Alam (2022) concluded that renewable energy is seen in innovations that can improve environmentally friendly technologies to reduce carbon emissions and enhance environmental quality.

We applied the PDOLS model to verify the consistency of the PFMOLS estimation. The findings from the PDOLS estimator are presented in Table 8. The findings present evidence of the robustness of the FMOLS model. Implicitly, the PDOLS outcomes confirmed the negative coefficients of agricultural growth on agricultural carbon emissions, although the results are now significant in model 3. Furthermore, the results of the PDOLS estimator verified the negative and statistically significant coefficients of renewable energy consumption at a 1% level of significance across both models. Interesting to note is that the outcomes of fertiliser consumption still mimic the same trend and pattern as those provide earlier. Moreover, the coefficients are still insignificant across all models. Similar to the results presented in the PFMOLS estimator, the improvements in the *R*^2^ to adjusted *R*^2^ values from the PDOLS model reflect the model’s goodness of fit.

**Table 8:** Results from the panel dynamic least square approach

Variable	1. None	2. Constant level	3. Linear trend	4. Quadratic trend
Agricultural growth	− 0.176 (0.154)	0.737*** (0.136)	− 0.249** (0.095)	− 0.538*** (0.122)
Fertiliser consumption	− 0.004 (0.018)	0.004 (0.027)	0.014 (0.021)	− 0.003 (0.019)
Renewable energy consumption	− 0.399*** (0.052)	− 0.583*** (0.086)	− 0.438*** (0.1008)	− 0.293** (0.105)
R-squared	0.592	0.941	0.984	0.989
Adjusted R-squared	0.338	0.898	0.970	0.978
S.E. of regression	18.8078	7.397	3.997	3.396
Long run variance	504.244	67.082	14.108	7.995

values in parenthesis are std errors. ***, **, and * at 1%, 5% and 10%, respectively

Source: authors’ calculation

## Discussion

The study investigates the nexus between agricultural carbon emissions, fertiliser consumption, agricultural growth and renewable energy consumption in Africa. The examined results of agricultural growth reveal a positive significant effect on agricultural carbon emissions in model 2 in both Table 7 and 8. The results corroborate the studies by Vo et al. (2020) and Zambrano-Monserrate et al. (2018), who found a positive association between GDP and carbon emissions. Our findings support those of (Raihan and Tuspekova 2022), who concluded that there is a positive correlation between GDP and carbon emissions in Peru. A rise in agriculture’s share of GDP is associated with deterioration in the environment through increased consumption and waste generation (Adebayo and Akinsola 2021). Measures to transition to a green economy in Africa will help save the continent from the impending global warming effects. The continent is still largely dependent on natural resources, with much of its population still depending on fishing, agroindustry and mining.

However, the results in model 4 of Tables 7 and 8 show a negative and statistically significant relationship between agriculture growth and carbon emissions from agricultural land-use change. This implies that an exponential growth in the agriculture sector’s share of GDP will lead to a reduction in the rate of carbon emissions. The exponential growth may afford African countries the opportunity to adopt best practices that have the potential to limit carbon emissions. The relationship between agriculture growth and carbon emissions is not linear but quadratic.

The results show a negative relationship between renewable energy consumption and carbon emissions. This shows the importance of renewable energy in mitigating the effect of carbon emissions in Africa. The results imply that increasing renewable energy in the total energy mix in Africa will help in reducing carbon emissions generated by agricultural land-use change. The results corroborate studies by Vo et al. (2020) and Zambrano-Monserrate et al. (2018), who concluded that there is a negative relationship between renewable energy consumption and carbon emissions. The results also support studies by Bilgili et al. (2016), Shafiei and Salim (2014), Paramati et al. (2017), and Bhattacharya et al. (2017), who found a significant negative relationship between renewable energy and carbon emissions.

However, this study has not established a statistically significant relationship between fertiliser consumption and carbon emissions in Africa, even though the results show positive signs. This outcome can be attributed to the low level of fertiliser utilisation in Africa.

## Conclusion/recommendations

The study examined the relationships between agriculture growth, fertiliser consumption, renewable energy consumption and agricultural carbon emissions in Africa for the period 1990 to 2019. A PFMOLS analysis was undertaken to determine whether their relationships were cointegrated in the long run or not. For a robustness check, a PDOLS analysis was also carried out. The results suggest that agriculture growth has a positive, statistically significant relationship with agricultural carbon emissions in the long run. The result of quadratic trend analysis, however, shows that, in the long run, agriculture growth has a negative statistically significant effect on agricultural carbon emissions. We found a negative statistically significant relationship between renewable energy consumption and agricultural carbon emissions in Africa. There was no statistically significant relationship between fertiliser consumption and agricultural carbon emissions. There was a unidirectional causality between agriculture growth and agricultural carbon emissions.

In brief, the agriculture sector must contribute to emissions reduction. If not, the Paris Agreement’s target of reducing CO_2_ emissions to 1.5 or below 2 degrees may not be achieved. Achieving an agricultural mitigation pathway for CO_2_ emission reduction requires a robust and integrated policy intervention approach (IPCC 2014).

In doing so, this study makes the following policy recommendations for policy and practice:

- First, we recommend adopting modern agricultural practices to reduce carbon emissions.
- Secondly, the government should make policies to incentivise farmers to adopt GHG-efficient and climate-smart farming technologies. This will not only reduce agricultural GHG emissions but also increase crop yield.

Last but not least, African countries should invest heavily in R&D for cost-effective renewable energy technology while aggressively updating existing production technology for green technology adoption in the agricultural-value chain. The growth in renewable energy may not only diversify the energy mix but also green as well.

We also recommend for future researchers to look at how specific farm practices at various sectors of agriculture contribute to agricultural carbon emissions in Africa.

## Data Availability

The data can be available at appropriate request.

## Appendix

Appendix Table 9

**Table 9:** Granger causality tests

Null Hypothesis:	Obs	F-statistic	Prob
AgricGDP does not Granger Cause AGREMIS	909	3.56920	0.0286
AgricEM does not Granger Cause AGRICGRW		2.04792	0.1296

## Appendix 2. Specification of unit root equations

4 $${\Delta Y}_{it}={\delta Y}_{it-1}+{\mu }_{it}$$

The equation for ‘with constant’ was stated as:

5 $${\Delta Y}_{it}=\alpha +{\delta Y}_{it-1}+{\mu }_{it}$$

The equation for ‘with constant and trend’ was stated as:

6 $${\Delta Y}_{it}=\alpha +\beta T+{\delta Y}_{it-1}+{\mu }_{it}$$

$${H}_{0}=\delta =0 (\mathrm{Unit root})$$

$${H}_{1}:=\delta \ne 0$$
